# Towards uniformly oriented diatom frustule monolayers: Experimental and theoretical analyses

**DOI:** 10.1038/micronano.2016.64

**Published:** 2016-12-05

**Authors:** Aobo Li, Wenqiang Zhang, Reza Ghaffarivardavagh, Xiaoning Wang, Stephan W. Anderson, Xin Zhang

**Affiliations:** 1 Department of Mechanical Engineering, Boston University, Boston, MA 02215, USA; 2 Department of Radiology, Boston University Medical Center, Boston, MA 02118, USA

**Keywords:** diatom, manipulation, monolayer, uniform

## Abstract

Diatoms are unicellular, photosynthetic algae that are ubiquitous in aquatic environments. Their unique, three-dimensional (3D) structured silica exoskeletons, also known as frustules, have drawn attention from a variety of research fields due to their extraordinary mechanical properties, enormous surface area, and unique optical properties. Despite their promising use in a range of applications, without methods to uniformly control the frustules’ alignment/orientation, their full potential in technology development cannot be realized. In this paper, we realized and subsequently modeled a simple bubbling method for achieving large-area, uniformly oriented *Coscinodiscus species* diatom frustules. With the aid of bubble-induced agitations, close-packed frustule monolayers were achieved on the water–air interface with up to nearly 90% of frustules achieving uniform orientation. The interactions between bubble-induced agitations were modeled and analyzed, demonstrating frustule submersion and an adjustment of the orientation during the subsequent rise towards the water’s surface to be fundamental to the experimentally observed uniformity. The method described in this study holds great potential for frustules’ engineering applications in a variety of technologies, from sensors to energy-harvesting devices.

## Introduction

It has been several decades since diatoms first drew the attention of researchers^1^. Diatoms are unicellular, photosynthetic algae notable for the remarkable exoskeletons enclosing their cytoplasm^2,3^. These exoskeletons, also known as frustules, are arranged in intricate hierarchical pore structures formed from amorphous biosilica. In some species, such as *Coscinodiscus species* (*C. sp.*) diatoms, the exoskeleton surfaces include up to three layers of pore structures, with pore sizes ranging from 60 nm to 1 μm. These hierarchical, porous structures give rise to the diatom frustules’ extraordinary mechanical strength^4^, enormous surface area ^5^, and unique optical properties^6,7^.

The development of the use of diatoms in a variety of technological applications is ongoing and, although abundant sizes and shapes of frustules are available, circular microscopic diatoms (such as *C. sp.*) are especially popular due to their complex hierarchical porous system and centrosymmetric structures^8,9^. To date, the use of diatoms as key components in a variety of application areas has been reported. For example, in applications to sensing technology, diatoms have been employed as the sensing components in immunoassays and gas sensors^10–14^. Furthermore, in work by Lin *et al.* ^11^, diatom frustules have been employed in electrochemical impedance sensing and have been found to significantly improve performance, including sensitivity, response time, and dynamic range, compared with existing approaches. In optical research, it has been demonstrated that diatom frustules behave as optical crystals, focusing light due to their micron and sub-micron scale pore arrays^15,16^. In addition, it has been reported that frustules have the unique ability to trap light due to their hierarchical pore structures^7,17,18^, a property that has been successfully leveraged in enhancing dye-sensitized solar cell performance^17^.

Given the 3D shape of diatom frustules, such as the commonly employed *C. sp.* frustules, the alignment and orientation of these biological materials is fundamental to optimizing their utility in a variety of applications. For example, in the application to biosensors, in which diatom frustules have been employed as bonding sites^19^, the ability to uniformly orient diatom frustules (especially in a ‘concave side facing down’ manner) may increase the utilization of the micro–nanostructures, thus increasing the bonding area. Moreover, in optical applications^7^, uniformly oriented frustules have the potential to form light focusing/trapping layers, thereby allowing more than just single diatoms to act as optical components, increasing the potential scale and efficiency of these devices. Importantly, the ability to manipulate the frustules on a large scale, combined with simple arraying techniques^20^, may enable the development of practical, real-world applications using diatoms. However, despite the promise of the use of diatom frustules in a variety of areas, the unique properties of diatoms have typically only been studied on the scale of a single diatom frustule (requiring only individual diatom alignment)^6–8,10–16^ or as bulk materials (requiring no significant alignment)^17^.

To arrange the diatom frustules into large-area monolayers, the use of self-assembly techniques may be considered. A variety of self-assembly approaches have been reported, including the assembly of particles with a range of sizes and shapes^21–23^. Special attention has been paid to the ‘floating assembly’ of micronanoparticles floating on liquid–air interfaces^24–26^, as this technique is technically relatively simple and produces high-quality monolayers or even 3D assemblies of particles. It has been reported that the success of the floating assembly results from the capillary forces between the floating particles^27,28^. Although initial progress has been reported in achieving the capacity for ‘batch control’ of frustule orientation as well as compactness as they form frustule monolayers on a flat substrate surface^29,30^, little attention has been paid to achieving uniform orientation in synchrony in large-area diatom monolayers. Herein, a universal method to obtain large-area, uniformly oriented diatom frustule monolayers is developed, with simulations and theoretical analysis conducted to illuminate the underlying principles.

## Materials and methods

### Diatom frustule preparation

Diatom frustules (*C. sp.*; catalog No. CCMP 1583, Bigelow Laboratory for Ocean Sciences, East Boothbay, ME, USA) were collected from 20 l of bulk culturing medium. The diatoms were initially settled and concentrated to a volume of 15 ml and subsequently cleaned with sulfuric acid. For this purpose, 350 ml of concentrated sulfuric acid was added to the condensed diatom culture medium; the mixture was then heated to 60 °C for 40 min. After cooling, the frustules were settled, and the supernatant of the solution was removed. The mixture was then added to deionized (DI) water for dilution and further settling. This dilution-settling procedure was repeated 4 to 5 times for further purification and concentration^31^ before the pH value of the solution reached neutral, after which the diatoms were stored in DI water. For subsequent imaging, the diatoms were first dried and dispensed onto clean silicon wafers, sputter coated with an ~10 nm gold/palladium alloy layer (Cressington 108 sputter coater, Cressington Scientific Instruments, Watford, UK) and then examined using a scanning electron microscope (Zeiss Supra 55VP, Zeiss, Thornwood, NY, USA).

### Uniformly oriented diatom monolayer formation

**Figure 1:**
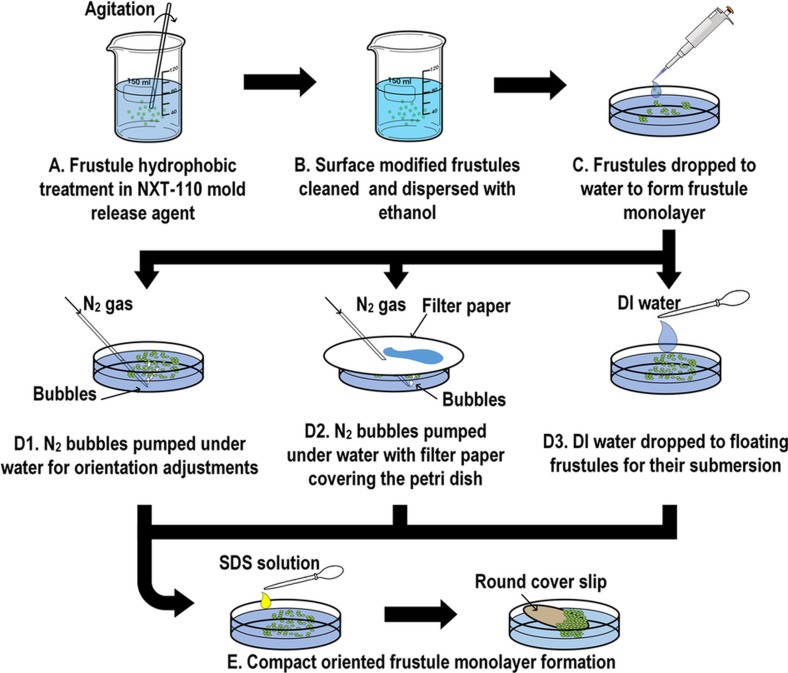
Process of uniformly arranging diatoms into a concave-down configuration. Three different approaches were employed to investigate the resultant concave-down frustule ratios (D1, D2, and D3).

To form compact, self-assembled monolayers of frustules on a water surface, the frustule surface was first rendered hydrophobic. For this purpose, 3 ml of NXT-110 mold release agent (Nanonex Corporation, Monmouth Junction, NJ, USA) were employed; after two parts of the agent (NXT-110 A & B, volume ratio A:B=1:2) were mixed, 0.1 g dry diatom frustules were immediately added to the solution. After 1 min of agitation, the frustules were filtered and rinsed with ethanol (Figure 1, A and B). The modified frustules were then dispersed in ethanol (weight ratio frustules: ethanol=1:10).

Hydrophilic thin glass cover slips (diameter ~13 mm) were prepared for the experiments involving the collection of the frustule monolayers by first soaking in 10% sodium dodecyl sulfate–water solution (SDS) for 2 h.

The prepared frustule suspension was slowly added, drop-by-drop, to DI water in a Petri dish with a diameter of 30 mm (Figure 1, C). The frustules floated on the water’s surface and formed a frustule layer. To adjust the frustule orientation, N_2_ bubbles were blown from ~3 mm beneath the water’s surface for 1 min for each group (Figure 1, D1). The N_2_ gas source was maintained at 5 psi and 0.1 SCFH throughout. The inner diameter of the N_2_ source nozzles was varied (0.076, 0.26, 0.52, 0.66, and 1 mm) to achieve different bubble sizes (see Results section). Statistics regarding the bubble sizes were obtained from slow-motion videos of the bubbling process. The bubble source was moved throughout the water-filled Petri dish to ensure agitation of the entire surface of the water by the N_2_ bubbles. To form a compact frustule monolayer, ~20 μl of 2% SDS solution was added to the water, and the frustules were compacted, forming a monolayer, which could then be lifted from the water’s surface using a modified cover slip (Figure 1, E). In the control groups, the floating diatoms were not agitated by the bubbles.

During this bubbling process, water jets from the rupturing bubbles were observed. Therefore, corresponding experiments were conducted to examine the effect of the water jets on the diatom frustule orientation. In these experiments, in addition to the previously described bubbling set-up, filter paper was used to cover the petri dish as N_2_ gas was pumped in (Figure 1, D2). In addition, in a control group, the experiments were performed without the filter paper. For these experiments, a nozzle diameter of 0.66 mm was used throughout.

In addition to the approach using N_2_ gas bubbles, an alternative agitation method was employed to further evaluate the interaction between water and diatoms. In this alternative method, the diatom frustules were initially dispersed along the water’s surface, forming diatom monolayers as described above. Subsequently, water droplets were released from 3 mm above the water’s surface, thereby impacting the floating diatoms. During this step, 2 ml pipettes with nozzle inner diameters of ~1.6 mm were employed, and water droplets with diameters of ~4 mm were generated. For each group of experiments, a total of ~2 ml of water was used to impact the floating diatoms along the water’s surface (Figure 1, D3). Following this alternative method of agitation, the frustules were collected using the SDS solution as described above for subsequent observation.

For each method described above, at least 4 groups of experiments were performed. The specimens were subsequently observed, and the relative numbers of frustules with the concave side facing upwards (concave-up) and downwards (concave-down) were counted using a scanning electron microscope, yielding the statistical data presented in the Results section.

### Analytical method for investigating the diatom-orienting phenomenon

Through careful observation of the bubble agitation procedure, we hypothesized that the disturbance of the water’s surface may have resulted in submersion of the frustules in water, followed by a floating (rising) of the diatoms back towards the water’s surface. Hence, using an analytical approach, we studied how submerged diatoms interacted with the surrounding water as they floated towards the water surface, focusing on the orientation of the diatoms during this procedure.

**Figure 2:**
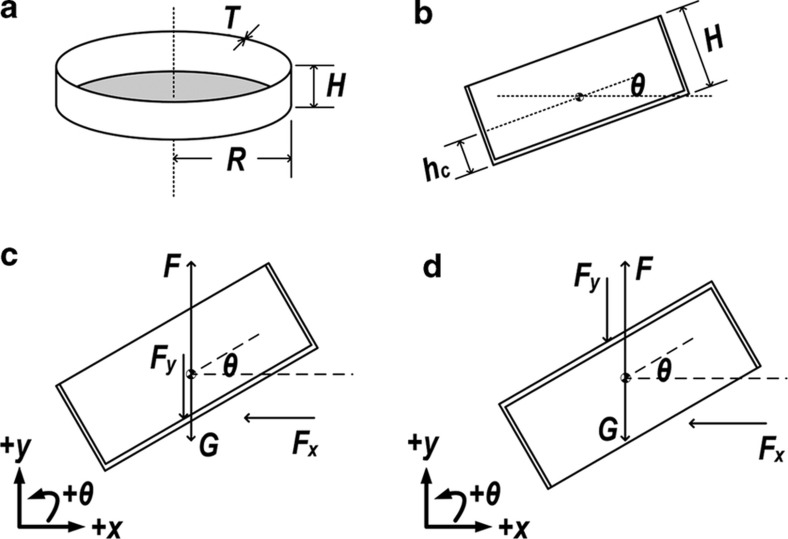
Theoretical analysis of diatom frustule interaction with water as it rises towards the surface following submersion. (**a**) An analytical model of a single diatom frustule. (**b**) A cross-section of the frustule model: *h*_c_ denotes the distance between the mass center and convex surface of a frustule. (**c**) Forces exerted on a rising ‘concave-up’ oriented diatom frustule: *F* denotes the buoyance force, *G* the gravitational force, *F*_*x*_ and *F*_*y*_ the drag forces resulting from the corresponding relative water flow. (**d**) Forces exerted on a rising ‘concave-down’ diatom frustule.

To this end, we developed a mechanical model of a submerged diatom frustule (Figure 2) with different initial conditions of depth and angular deviation (*θ* in Figure 2). We sought to use mechanics approaches to analyze a simplified model of a frustule and provide a qualitative description of the propagation of the frustule’s horizontal movement (*x*(*t*)), vertical movement (*y*(*t*)), and angular deviation (*θ*(*t*)). An increasingly accurate quantitative description of the frustule’s movement may also be derived using numerical methods, which will also be discussed in this paper.

For theoretical analysis and the subsequent simulations, a reduced 2D model of the diatom frustule was built (Figures 2a and b, Supplementary Materials, Supplementary Figure S1). The known conditions of the model and the assumptions are stated as follows. According to our previous observations of the *C. sp.* diatom frustules, the radius (*R*), height (*H*), and thickness (*T*) of a *C. sp.* diatom frustule were set to 61, 17.15, and 1 μm, respectively. The frustules’ effective density approximates 120–250 kg m^−3^ (Ref. 32); for the computation, we employed a density of 150 kg m^−3^. This geometric model was employed to estimate its related parameters, including mass (*m*), position of mass center (*h*_c_; Figure 2b), and moment of inertia (*I*). To avoid the zero-thickness effect of a 2D model, we also introduced a virtual out-of-cross-section-plane ‘depth’ of the frustule (*d*; Supplementary Materials, Supplementary Figure S2).

A frustule in water experiences gravitational force (*G*, as shown in Figure 2), buoyancy force (*F*), and drag forces from the interaction between the diatom and its surrounding water (*F*_*x*_ and *F*_*y*_; Figures 2c and d). The drag force is exerted against the direction in which the diatom is moving and is mainly due to the momentum change of the water moving in *x* and *y* directions. Due to viscosity and the frustule’s acceleration and deceleration in water, added mass (*m*_a_) and added momentum of inertia (*I*_a_)^33,34^ are also considered in this analytical model. Note that the movements of water in both the *x* and *y* directions against the frustules will generate component forces along the *x* and *y* directions.

For a diatom with its concave side initially facing upward, the force balance equations in the *x* and *y* directions are as follows:

(1)∑Fx=−Fxx+Fxy=(m+ma)x''(t)∑Fy=F−Fyy+Fyx−(m+ma)g=(m+ma)y''(t)

where *x*″(*t*) and y″(*t*) represent the frustule’s acceleration along the *x* and *y* directions, respectively, and *F*_*ij*_ stands for the component force in the *i* direction resulting from water flow in the *j* direction. The forces on the left hand side of Equation (1) can be expressed as follows:

(2)Fxx=2ρRd[x'(t)]2sin3θ(t)+ρHd[x'(t)]2cos3θ(t)Fxy=ρd[2R−Htanθ(t)][y'(t)]2cos2θ(t)sinθ(t)−2ρHd[y'(t)]2sin2θ(t)cosθ(t)F=ρVdgFyy=ρd[2R−Htanθ(t)][y'(t)]2cos3θ(t)+2ρHd[y'(t)]2sin3θ(t)Fyx=2ρRd[x'(t)]2sin2θ(t)cosθ(t)−ρHd[x'(t)]2cos2θ(t)sinθ(t)

where *ρ* is the density of water, *x*′(*t*) and *y*′(*t*) are the frustule velocities in the *x* and *y* directions, respectively, *V_d_* is the volume of the diatom frustule, and *g* is the gravitational acceleration. The initial tilt angle (*θ*(0)) of the diatom frustule with respect to the horizontal plane can be set to varying values, and *θ*(0)=0–90° was defined as corresponding to the case in which the diatom begins rising with its concave side facing upwards.

To develop a rotational model of the diatom, we assume that the drag forces are uniformly distributed along the surface of the frustule (Supplementary Material, Supplementary Figure S1). Note that there is a small volume within the frustule (marked as green in Supplementary Figure S1) that is not under the influence of this force, as it is shadowed by the sidewall of the frustule. By summing the torque from all parts of the frustule with respect to the mass center, we derive the total torque and the rotational movement model of the frustule as follows:

(3)∑M=Mx+My=(I+Ia)θ″(t)

where *M*_*x*_ and *M*_*y*_ are the torques resulting from the drag forces *F*_*x*_ and *F*_*y*_, and *θ*″(*t*) is the angular acceleration of the frustule. The torques can be further expressed as follows:

(4)Mx=ρHd[x′(t)]2cos2θ(t)(H2−hc)My=2ρHd[y′(t)]2sin2θ(t)(H2−hc)+ρd[2R−Htanθ(t)][y'(t)]2cos2θ(t)Htanθ(t)2

Equations (1) and (3) are coupled, and by solving them together, the linear and rotational movements of the frustule can be determined. Equations (1) and (3) are also appropriate in the case of a frustule with its concave side initially facing downward; however, in this case, the component forces and torques are changed as follows:

(5)Fxx=ρd[2R−Hcotθ(t)][x′(t)]2sin3θ(t)+2ρHd[x′(t)]2cos3θ(t)Fxy=2ρRd[y′(t)]2cos2θ(t)sinθ(t)−ρHd[y′(t)]2sin2θ(t)cosθ(t)Fyy=2ρRd[y′(t)]2cos3θ(t)+ρHd[y′(t)]2sin3θ(t)Fyx=ρd[2R−Hcotθ(t)][x′(t)]2sin2θ(t)cosθ(t)−2ρHd[x′(t)]2cos2θ(t)sinθ(t)Mx=−2ρHd[x′(t)]2cos2θ(t)(H2−hc)−ρd[2R−Hcotθ(t)][x′(t)]2sin2θ(t)Hcotθ(t)2My=−ρHd[y′(t)]2sin2θ(t)(H2−hc)

The magnitudes of the added mass and added momentum of inertia were calculated from the reported formula for a rectangular cross-section for in-plane translation and rotation^34^. In this case, *θ*(0)=0–90° was defined as corresponding to the case in which the diatom begins rising with its concave side facing downwards. Finally, the equations were solved numerically using the Runge–Kutta method. The detailed derivation of the forces can be found in the Supplementary Material.

### Diatom submersion and motion simulations

To more fully understand the underlying factors resulting in the change in orientation of the diatom frustules following N_2_ bubble agitation of the water’s surface, simulations were conducted to analyze the interaction between the water surface agitation and the floating diatoms. To this end, two steps were undertaken to simulate the interactions among the N_2_ bubbles, water, and diatom frustules: the first step was to analyze the process of the diatoms’ submersion into water, and the second step was to analyze the process of the diatom frustules floating back towards the water’s surface following submersion. Of note, the simulations of the diatom’s rise towards the water’s surface address the same problem for which we sought solutions in the analytical method detailed above, thereby serving to verify both the experimental results and the analytical solutions, as well as facilitating a more comprehensive understanding of this phenomenon.

The process of a bubble rupturing was initially simulated using COMSOL (Supplementary Material, Supplementary Figures S3–S5). A cylindrical tank with a diameter of 32 mm, a 10 mm depth of water, and a 10 mm layer of overlying N_2_ was employed for the simulation, which corresponded to our experimental setups. N_2_ bubbles ranging in size from 3.2 to 4.2 mm in diameter were initially simulated as originating 6 mm beneath the water’s surface prior to release, and a time scale of 0.5 s was set to record the rupturing of the bubbles. The displacement of the water’s surface induced by the rupture of the bubbles, was analyzed at a distance of 3 mm from the center of the rupturing bubble.

To verify the influence of the ruptures on the diatoms and analyze the diatoms’ interaction with the water, a smoothed particle hydrodynamics (SPH) method was used^35^. In this method, to reduce the amount of calculation, a cross-section of the water-diatom model (a 2D model) was simulated and analyzed. To simulate the effect of the rupturing bubble on the diatoms, a water tank of 3 mm in width and 0.8 mm in depth was simulated. A wave generator was simulated along one end of the tank to produce water surface agitation. Importantly, the water displacement data from the previous simulation were used as the motion data for the wave generator, which consequently mimics the water agitation following bubble rupture. A 2D model of a single *C. sp.* frustule was also built according to the aforementioned parameters. In the simulation, the frustule was placed 1 mm from the wave generator to avoid direct contact between these structures.

To simulate the interactions between the diatom frustule and the water following submersion, COMSOL models of a single diatom frustule being released from different depths beneath the water’s surface (100, 200, 300, 400, and 500 μm) were used. A frustule traveling through the water–air interface was also simulated. For both types of simulations, the same geometrical parameters and material properties of the frustules and water were used as in the analytical calculations.

In the frustule-release simulations, the COMSOL fluid-structure interaction (FSI) module was used. The governing equations for the incompressible fluid phase are the following Navier–Stokes equations of momentum and continuity:

(6)ρ(∂u∂t+u⋅∇u)=∇⋅[−pI+µ(∇u+(∇u)T)]+f∇⋅(ρu)=0

where *ρ* is the density of the fluid (in our case, water), **u** the velocity field of the fluid, *p* the pressure, *μ* the dynamic viscosity, and **f** the external source term. Both the solid (frustule) and fluid (water) phases were subject to gravity.

In the interactions between the solid and fluid, the total forces exerted on the solid’s surface are equal to the force on the fluid:

(7)FT=n⋅[−pI+µ(∇u+(∇u)T)]

Except for the boundary load exerted on the solid’s surface, the fluid-structure boundary conditions of equal displacement and velocity were applied through a moving mesh. The frustule–water interactions were calculated under the influence of drag force, buoyancy force, and gravity.

To reduce the calculation load, a 2D model was constructed. In the FSI module, a deformable moving mesh was used for fluid domain calculations. In our application, the free travelling frustule structure may cause large deformations of the mesh, and therefore, automatic remeshing was also employed for solving the models.

To examine the process of a frustule travelling through the water–air interface, another set of simulations was conducted. Since the process involves the interaction among three phases (solid, gas, and liquid), a two-phase fluid model was used. A phase field approach was employed in COMSOL to track the interface between the fluid phases^36,37^. This method is based on tracking the mixing energy at the interface. Due to defining a phase field variable *ϕ*(*ϕ*=±1) for water and air, there will be an additional governing equation for calculating the evolution of *ϕ* at the interface.

(8)∂ϕ∂t+u⋅∇ϕ=∇⋅[γλε2∇ψ]ψ=−∇⋅ε2∇ϕ+(ϕ2−1)ϕ

Here, *γ* is the mobility, *λ* is the magnitude of the mixing energy, and *ε* is a capillary thickness^36^. Together with the aforementioned governing equations, boundary conditions, and moving mesh/automatic remeshing conditions, the three-phase interaction problem was solved.

## Results

### *Coscinodiscus species* frustule morphology

**Figure 3:**
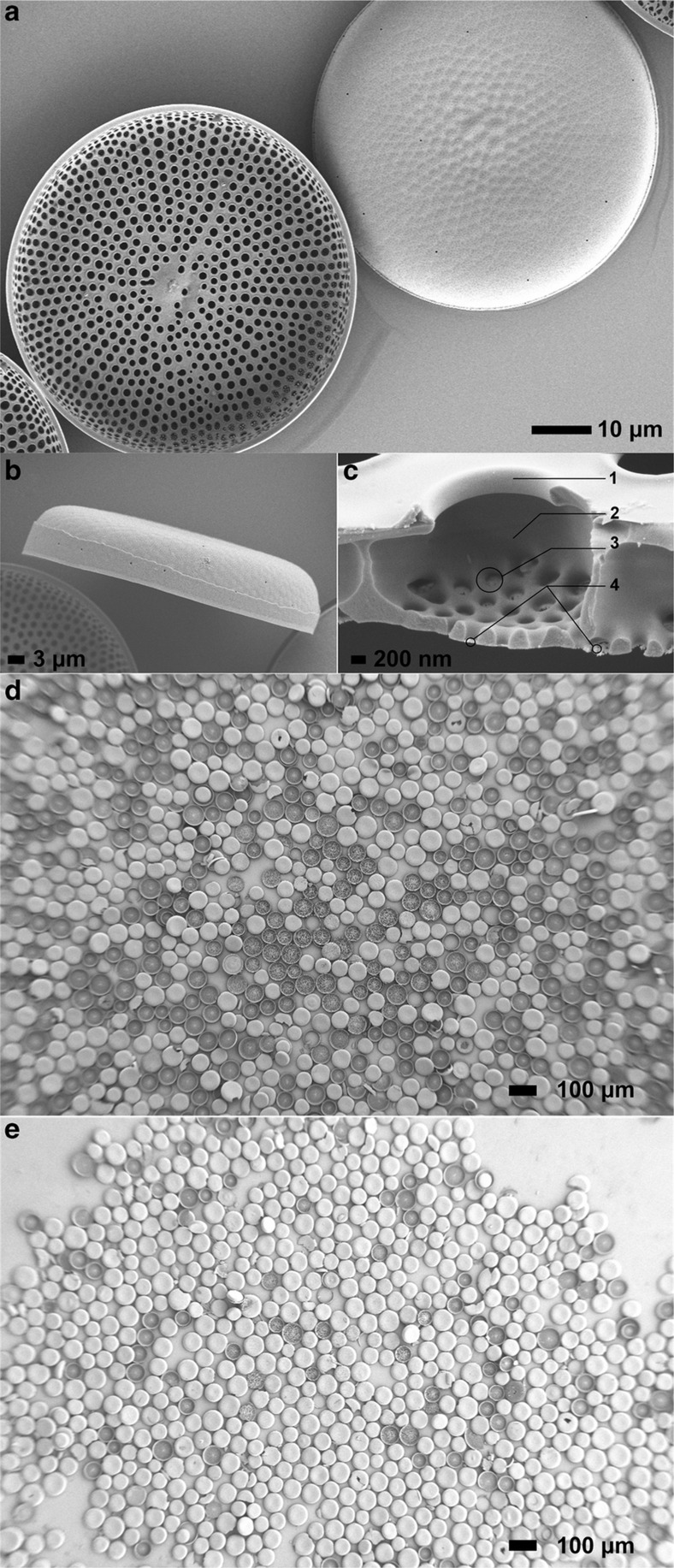
*C. sp.* diatom frustules following sulfuric acid cleaning and monolayer assembly. (**a**) A frustule with its concave side facing upwards (lower left) and a frustule with its concave side facing downwards (upper right). (**b**) Side view of a single *C. sp.* diatom frustule. (**c**) Cross-section of a *C. sp.* frustule: labels 1–4 indicate different porous layers from the concave side to the convex side of a frustule, where 1 shows a partial foramen pore, 2 shows an areola chamber wall, 3 shows a cribrum pore, and 4 shows the cribellum pores. (**d**) Diatom monolayer without bubble-induced agitation: ~60% of the diatoms were in a ‘concave-down’ orientation. (**e**) Nearly 90% of the diatoms were successfully adjusted to a ‘concave-down’ orientation on a substrate after bubble-induced agitation.

As noted above, the *C. sp.* diatom frustules shown in Figures 3a and b are dish-like, circular micron-scale valves, the diameter of which ranges from 50 to 150 μm. The total height of a *C. sp.* frustule is ~12 μm. Along the concave surface of the frustules, micropores (termed foramen) with diameters ~1 μm are evenly distributed, while on the opposite, convex side, nanopores (termed cribellum) with diameters ranging in size to a minimum of 60 nm form the frustule surface. Between the foramen and cribellum pore layers, a layer featuring sub-micron pores (termed cribrum) is found in *C. sp.* diatom frustules (shown in Figure 3c) with diameters of ~200 nm. The microchambers enclosed by the foramen pore layer, the cribrum–cribellum layer, and the frustule side walls are termed areolae. The overall thickness of a single valve of the *C. sp.* diatom frustules consisting of the aforementioned hierarchical pore structure is approximately 1–1.2 μm.

### Uniformly oriented diatom monolayer formation

**Figure 4:**
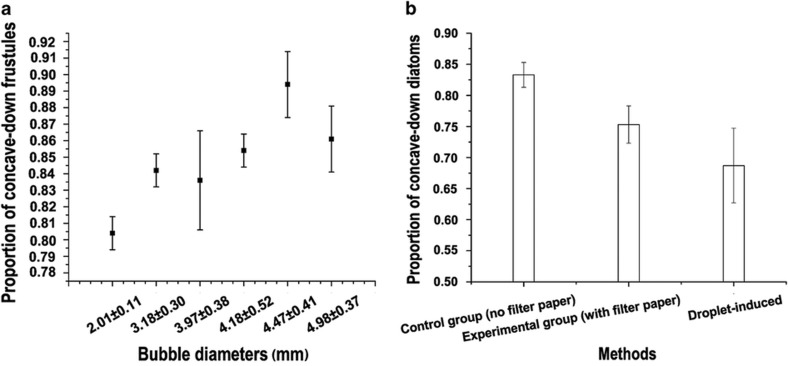
Proportion of concave-down diatoms as a function of different bubble sizes with the N_2_ bubbling method (**a**) and proportion of concave-down diatoms as a function of different agitation methods (**b**).

Following surface modification with the release agent, the diatoms possessed a decreased surface energy, and their wetting became increasingly difficult. We hypothesize that this change allows the hierarchical pore and microchamber system to trap air within the frustule substructures, leading to the observed flotation of the frustules. After initial dispersion along the water’s surface, compact diatom frustule monolayers can be formed with or without bubble agitation (Figures 3d and e) before adding SDS solution to the water’s surface. Note that upon addition of the SDS solution to the water’s surface, the surface tension at the entry point of the SDS solution dropped instantaneously, while the tension of other areas of the surface remained static at that particular moment. This effect resulted in a contraction of the water’s surface at higher tension, including the floating frustules, thereby yielding a compact monolayer in a short time. The area of the monolayer without bubble agitation ranged in size up to 1.1 cm^2^, nearly covering the entire surface of a cover slip, whereas the monolayer area after bubble-induced agitation decreased slightly, ranging in size up to 0.8 cm^2^. We found that by using the N_2_ bubble-induced agitation approach, up to nearly 90% of diatom frustules were found to orient with their concave side facing downwards (concave-down), although in the control group, 58.8±1% of the diatoms were adjusted to a concave-down orientation. It was hypothesized that the agitation from the rupturing of the N_2_ bubbles was the predominant factor affecting the orientation of the diatom frustules (concave-up versus concave-down) floating on the water’s surface. Therefore, the correlation between the orientation of the diatoms and the agitation amplitude was analyzed using bubbles of varying size. The evaluation of the frustule orientation as a function of N_2_ bubble size is shown in Figure 4a, revealing that generally, as the bubble size increased, the concave-down diatom ratio increased.

By adding a layer of filter paper, we observed that during the bubble-induced agitation, the generated water jets from the bubbles wetted the paper. As the procedure continued, water jets were consecutively absorbed by the filter paper. In this manner, some of the water jets were prevented from falling back towards the water’s surface, mitigating their effect on the frustules along the water’s surface. We found that 75.3±3% of the frustules were adjusted to a concave-down orientation in these experiments, although in the case of the control group without the addition of the filter paper, 83.3±2% of the frustules were adjusted to a concave-down orientation (Figure 4b). These results demonstrate that the water jets induced by the bubble rupture are likely to have a role in the final frustule orientation.

With the alternative method of diatom frustule agitation using the water droplet-based method, droplets were found to impact and submerge the majority of the floating diatoms. After the initial submersion, the majority of the diatoms would rise and again reach the water’s surface. Following submersion of the diatoms and their subsequent rise towards the water’s surface in this fashion, it was found that 68.7±6% of the diatoms were adjusted to a concave-down orientation.

### Analytical and simulation analyses of diatom orientation

**Figure 5:**
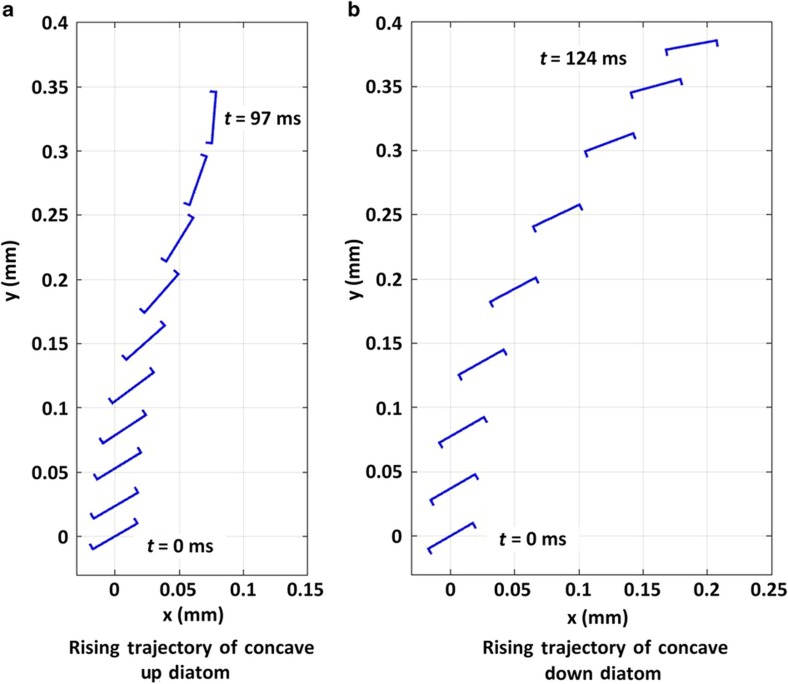
Theoretical solutions of diatoms rising towards the water’s surface following submersion, as a function of varying initial orientation. (**a**) A concave-up frustule’s rising trajectory. (**b**) A concave-down frustule’s rising trajectory. The initial tilt angle (*θ*(0)) of both frustules is 30°.

Using the analytical model we built and described above, the diatom frustule motion (*x*(*t*), *y*(*t*) and *θ*(*t*)) as a function of time may be solved for different initial angular deviations (*θ*(0)). Figure 5 demonstrates the calculated movements of concave-up and concave-down diatom frustules. In both cases, *θ*(0)=30° for demonstration purposes. It is evident from Figure 5a that through the adjustment of the drag force exerted on the diatoms, a concave-up diatom can be tilted to increase its angle with respect to the horizontal plane. The frustule’s tilt angle, *θ*(*t*), will eventually reach π/2 and beyond, flipping the frustule. Thus, through this motion, the initially concave-up frustule will change its orientation to a concave-down configuration. However, in the case of the initially concave-down configuration, the adjusting drag force does not induce an increase in the tilt angle (Figure 5b). Ultimately, these frustule motions will result in a concave-down orientation during the rise towards the water’s surface following submersion.

To verify the experimental and analytical results, simulations were performed to study the frustules’ behavior after N_2_ bubble rupture. It was found that the bubbles’ rupturing generated waves with amplitudes ranging from 0.7 to 2.5 mm in size (Supplementary Material, Supplementary Figure S3), whereas the sizes of the frustules ranged from 50 to 150 μm. Given waves of this size, some of the diatom frustules were readily submerged beneath the water’s surface (Supplementary Video). When submersion was not achieved, the frustule was found to be moved violently and randomly along the water’s surface.

**Figure 6:**
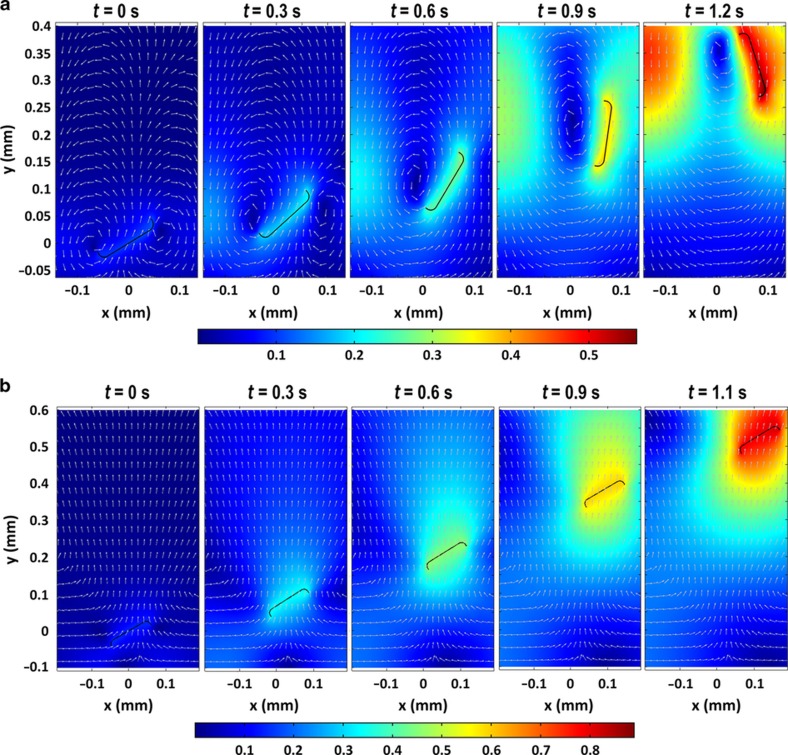
Simulated rising 2D diatom frustule and corresponding velocity field (unit m s^−1^) of surrounding water. The initial tilt angle (*θ*(0)) of both frustules was 30°. (**a**) The rising of a concave-up frustule. The frustule went through a self-adjusting process and eventually reoriented to a concave-down configuration. (**b**) The rising of a concave-down frustule. The tilt angle of the frustule oscillated between 29.9 and 32.6° throughout the process.

The COMSOL simulations showed a similar behavior to the analytical approach. Once submerged, the frustule initially travels with the underlying water currents and eventually floats back towards the water’s surface, self-adjusting to a concave-down orientation during this process (Figure 6).

**Figure 7:**
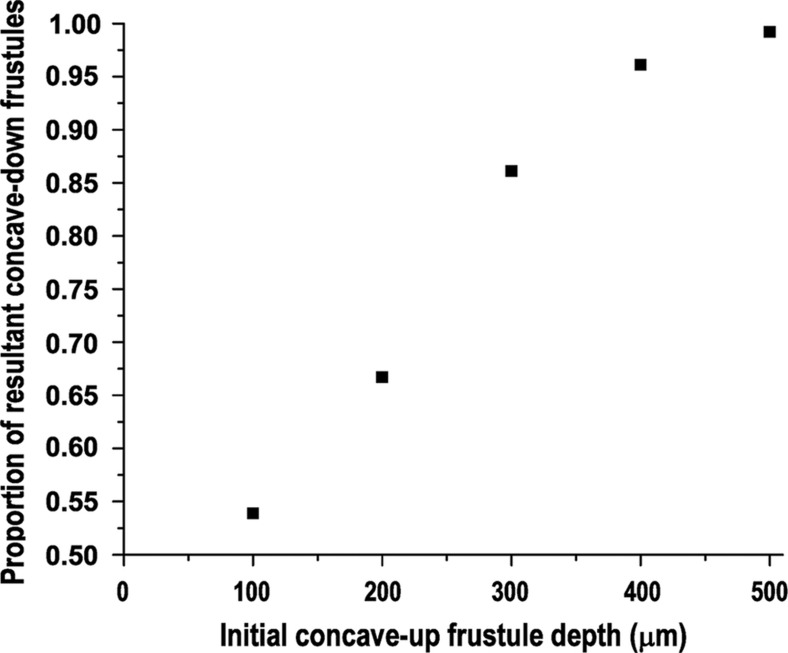
Simulation results of the influence of the initial depth of a concave-up diatom frustule on the final concave-down ratio.

Assuming an equal chance of any initial tilt angle (*θ*(0)) after being submerged, we can calculate the corresponding proportion of diatoms that could eventually reach the critical angle of π/2. In the COMSOL simulations, an initial depth of 100 μm was first analyzed with varying initial tilt angles (*θ*(0)). When *θ*(0) was ⩾83° (corresponding to 53.9% of all submerged diatoms), the diatoms’ *θ* became π/2 as they rose to the water’s surface. When the depth was increased to 200 μm, 66.7% were adjusted to π/2. Furthermore, when the initial depth was 300, 400, and 500 μm, 86.1, 96.1 and 99.2% of all submerged diatoms, respectively, were adjusted to π/2, indicating that, as the submersion depths of the diatoms increased, increasing numbers of diatom frustules would orient towards a concave-down configuration (Figure 7).

**Figure 8:**
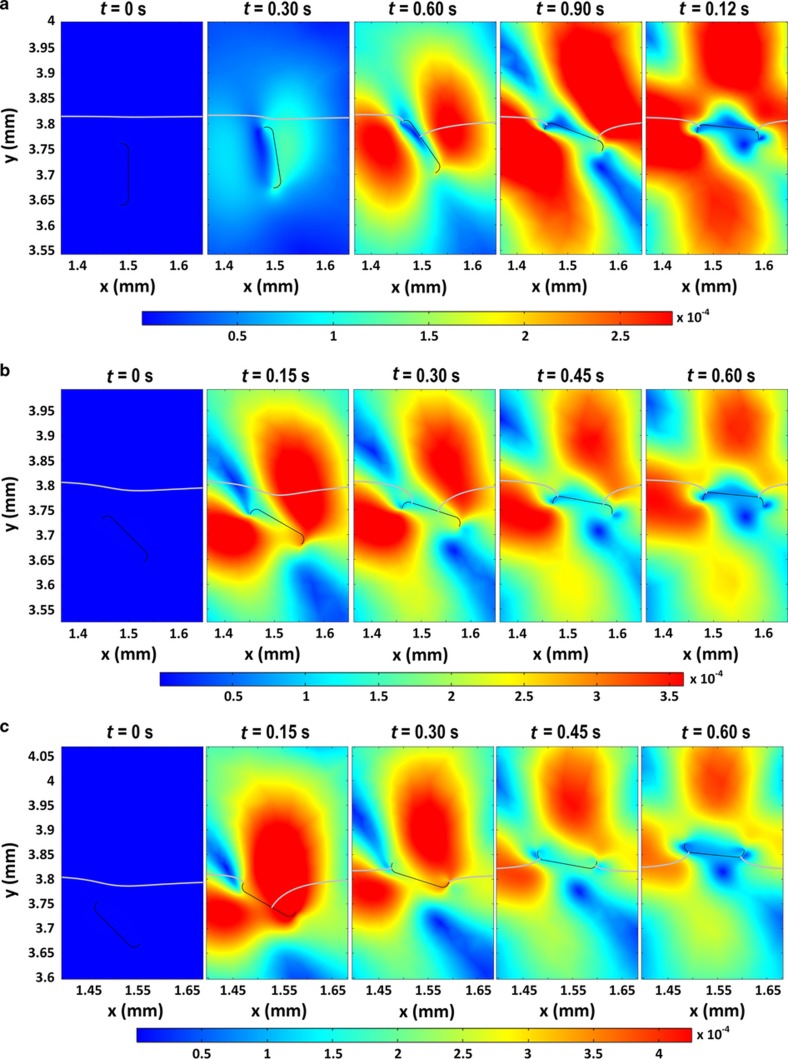
Frustules breaching the water–air interface and corresponding velocity field (unit m s^−1^). Gray curves indicate the water–air interface. All frustules were released with 0 velocity. (**a**) The initial tilt angle (*θ*(0)) of the frustule was 90°. (**b**) The rising of a concave-down frustule with an initial angle of 45°. In (**a**) and (**b**), both frustules passed through the interface and eventually reoriented to a concave-down configuration. (**c**) The rising of a concave-up frustule with an initial angle of 45°, which ultimately ended on the water’s surface with a concave-up configuration.

For the solid–liquid–gas interactions, we are mainly interested in determining whether the frustules that have already reached π/2 will eventually breach the water-air interface and continue to roll over to assume a concave-down configuration. We are also interested in whether frustules with a concave-down or concave-up configuration will maintain their posture as they breach the water-air interface. As illustrated in Figure 8, both the frustule with *θ*(0)=90° and the concave-down frustule with *θ*(0)=45° finally breached the water–air interface as they reoriented themselves to a concave-down configuration. However, the concave-up frustule with *θ*(0)=45° arrived at the surface with a concave-up configuration. The simulated phenomenon indicated that as diatoms travel through the water’s surface, the interaction among the frustule, water and air will not suddenly change the turning direction of the frustules.

## Discussion

This study reports that through the process of N_2_ bubble-induced agitation, diatom frustules achieved a highly uniform concave-down orientation. Subsequently, we sought to examine and more completely understand this phenomenon through experimental and analytical inquiry, along with simulations.

We first conducted additional experiments using filter paper to mitigate the effects of bubble rupture-induced water jets interacting with the diatom frustules. We hypothesize that the decrease in concave-down frustule ratio achieved in this fashion is due to a less efficient submersion of diatom frustules, with submersion being fundamental to the capacity of the frustule to reorient in the concave-down orientation. In addition to the analysis of the bubble agitation method, a water droplet-induced agitation method was also employed, and the results also corresponded well with the subsequent analytical and simulation results—nearly 70% of the diatoms were reoriented to a concave-down configuration. In addition, in the COMSOL simulations of rupturing bubbles, water jets (Supplementary Materials Supplementary Figure S4) and sinking currents (Supplementary Materials Supplementary Figure S5) were observed near the bubbles after they burst. Prior studies^38^ have also discussed the possible influence of such rupturing upon floating objects, reporting that when the radius of the bubble is greater than a floating ‘ship’ and when the floating ‘ship’ is positioned close to the outer rim of the bubble when it reaches the water’s surface, it is possible for the bubble to cause the ‘ship’ to sink, lending further support to our conclusion that submersion is a major contributing process in adjusting the diatoms’ orientation. We thus conclude that the predominant effect of the bubble rupture is the creation of surface agitation, leading to submersion of the diatom frustules, and that this submersion is fundamental to the effect on the diatom uniformity observed using N_2_ bubble-induced water surface agitation.

When comparing the water droplet-induced agitation with the bubble-induced agitation, however, fewer water droplets than bubbles were generated during a given period of time. In addition, since the water droplets were generated individually and manually, the surface area of the agitation was limited at any given time, although the continuous motion of the bubble-induced agitation affected the entire surface of the water continuously. We hypothesize that these differences between the methods of water surface agitation likely resulted in the observed smaller flipped-diatom ratio of the water droplet method compared with the bubble method.

To summarize, based on the experimental results, we conclude that after bubble rupture, diatoms are submerged beneath the water’s surface and undergo a series of interactions with water. Water drag alters the orientation of the submerged diatoms, and due to the imbalance in forces exerted on the frustule, a concave-up frustule rising towards the water’s surface is found to eventually reorient into a concave-down configuration. However, as a concave-down frustule rises in the water following submersion, no reorienting occurs, eventually resulting in the frustule floating on the water’s surface in a concave-down state.

In this paper, the analytical model serves as a qualitative description of the frustule’s rising in water, given the requisite simplifications employed for the ease of calculations. The discrepancy between the analytical and simulation results can be attributed to the different methods employed to analyze the model. In the analytical approach, we employed momentum change and added mass to account for drag forces and viscous forces exerted on the diatom frustule body, both of which reflect simplifications of the fluid-structure interaction. However, in the simulation model, pressure distribution and viscous stress were evaluated, yielding more accurate predictions of the fluid-structure interaction.

The numerical method is applied for qualitative analysis, noting that it represents a simplified model; the dish-like 3D structure and the micro/nanopores of the diatom frustules are not considered. Nevertheless, the analytical solutions and simulations provide a degree of insight into the likely behavior of the submerged frustules. They indicate that, given a sufficient rising distance (following a sufficient submersion depth), all diatoms may reorient towards a concave-down configuration on the water’s surface following submersion. Given the combined insights derived from the analytical and simulation results, we conclude that the interaction between rising diatoms and water is a significant factor in determining the ultimate orientation of the diatoms.

The analytical and simulation results yield useful initial insight into the frustule agitation/submersion process and address the experimentally observed uniform orientation phenomenon of the *C. sp.* frustules. However, the nature of the agitations, due to the rapid and consecutive rupturing of bubbles, means that the direct recording and analysis of the diatoms’ submersion and subsequent motion during their rise towards the surface is highly technically challenging. Additional experimental improvements such as directly releasing modified diatoms in water at precisely controlled submersion depths will allow us to more closely scrutinize the validity of the analytical and simulation results with respect to the experimental findings. Furthermore, high-speed imaging techniques have the potential for direct investigation of the interaction between the diatom frustules and the rupturing bubbles.

## Conclusion

In this paper, an experimentally observed phenomenon of *C. sp.* frustules orienting preferentially in a specific, concave-down manner following agitation by N_2_ bubbles is reported. It was found that following bubble agitation, up to nearly 90% of the frustules were arranged into monolayers with their concave sides facing downward. Furthermore, it was revealed that with an increase in the bubble size employed during bubble-induced agitation, the concave-down diatom ratio was increased. It was concluded from both analytical study and simulations that the concave-down orientation was ultimately favored following the diatoms’ rise towards the water’s surface after submersion. The method in this paper has the potential to evolve into a relatively convenient fabrication procedure for batch arranging the frustules in a uniform orientation. The capacity to manipulate diatoms in such a manner could be employed in furthering a variety of biologically enabled technologies, from sensor fabrication to light-trapping energy applications. Beyond the specific case of diatom frustule orientation examined herein, the convenient agitation method employed to adjust a number of asymmetrically shaped micro particles on the water’s surface, along with the corresponding analytical and simulation models, may be generalized and could potentially be beneficial for the manipulation of myriad asymmetrically shaped micro devices/particles.
