# Supplementary material for: Towards uniformly oriented diatom frustule monolayers: Experimental and theoretical analyses
Source: Microsyst Nanoeng. 2016 Dec 5;2:16064. doi: 10.1038/micronano.2016.64 (PMC6444733; doi:10.1038/micronano.2016.64)
Supplement: Supplementary Information [file micronano201664-s1.pdf]

Supplementary file

Towards uniformly oriented diatom frustule monolayers: Experimental and theoretical analyses

Aobo Li[1], Wenqiang Zhang[1,*], Reza Ghaffarivardavagh[1], Xiaoning Wang[1], Stephan W. Anderson[2] and Xin Zhang[1]

Microsystems & Nanoengineering (2016) **2,** 16064; doi:10.1038/micronano.2016.64; Published online: 5 December 2016

DERIVATION OF DIATOM FRUSTULES' MOVEMENT IN WATER

In this analysis, the freely travelling diatom frustule experiences drag forces from both the x and y directions. The drag force exerted on a frustule is due to the momentum change in water. Here we have:

$$F\Delta t = m_w \Delta v$$

$$F = \dot{m}_w \Delta v$$

where Δt denotes time, m_w denotes mass of water, Δv denotes the change in the water's relative velocity with respect to diatom frustule and $\dot{m}_w$ is the mass flow rate of water flow.

By taking a concave-up diatom frustule as an example, its drag forces and the resultant torques that are being exerted on it will be analyzed. As shown in Figure S1, we first consider the component force in the y direction resulting from y direction water flow.

According to Figure S1, the shaded part (green) is not being affected by water flow and, therefore, this portion is excluded during the calculations, yielding the effective mass flow rate in the y direction (a derivation of the effective diatom depth d will be shown later):

$$\dot{m}_{wy} = \rho d[2R \quad H\tan\theta(t)]\cos\theta(t)[y'(t)] + 2\rho Hd\sin\theta(t)[y'(t)]$$

where the first term represents the water flow that affects part 1 in Figure S1, and the second term represents the water flow that affects part 2 and part 3.

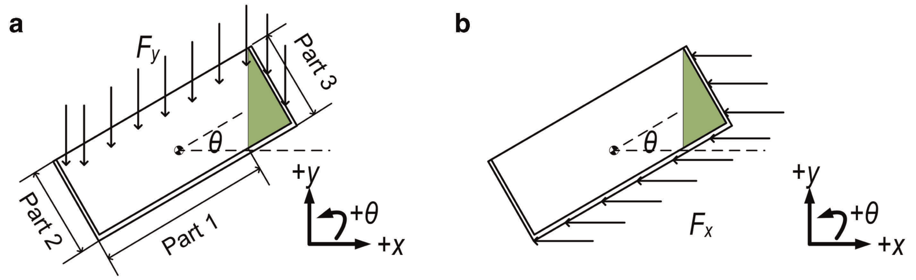

Figure S1 Theoretical analysis of a concave-up diatom rising in water. (**a**) The *y* direction analyses. (**b**) The *x* direction analyses.

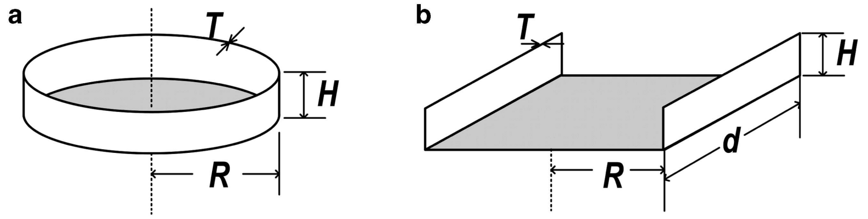

Figure S2 Simplified model of a diatom frustule (**a**) and an equivalent model of a frustule (**b**).

[1]Department of Mechanical Engineering, Boston University, Boston, MA 02215, USA and [2]Department of Radiology, Boston University Medical Center, Boston, MA 02118, USA.
Correspondence: Stephan W. Anderson (Stephan.Anderson@bmc.org) or Xin Zhang (xinz@bu.edu)
*Present address: College of Engineering, China Agricultural University, Beijing 100083, China.

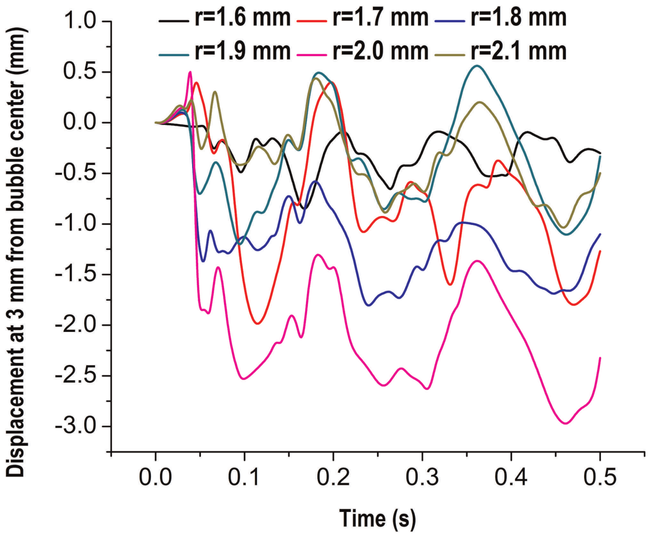

Figure S3 Water surface displacement after different sized bubbles rupture.

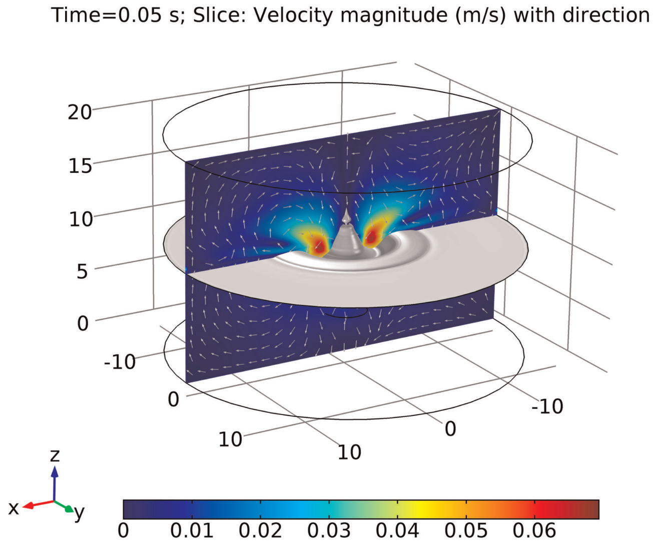

Figure S4 Velocity field of simulated rupturing bubble. Water jet generated during this process can be clearly seen in the figure.

The force that is being exerted perpendicular to parts 1-3 may be written as:

$$F_{\text{Part1}} = \{\rho d[2R \quad H\tan\theta(t)]\cos\theta(t)[y'(t)]\}[y'(t)]\cos\theta(t)$$
$$= \rho d[2R \quad H\tan\theta(t)][y'(t)]^2\cos^2\theta(t)$$

$$F_{\text{Part2+Part3}} = \{2\rho Hd\sin\theta(t)[y'(t)]\}[y'(t)]\sin\theta(t)$$
$$= 2\rho Hd[y'(t)]^2\sin^2\theta(t)$$

and the sum of their y components yields F_{yy} :

$$F_{yy} = \rho[2R \quad H\tan\theta(t)]d[y'(t)]^2\cos^3\theta(t) + 2\rho Hd[y'(t)]^2\sin^3\theta(t)$$

Similar analyses may be conducted to achieve all other component forces.

Torque that is being exerted on the frustule due to the y direction water flow can be derived as:

$$M_{F_{\text{Part1}}} = \rho d[2R \quad H\tan\theta(t)][y'(t)]\cos^2\theta(t)\frac{H\tan\theta(t)}{2R \quad H\tan\theta(t)}\left[R \quad \frac{H\tan\theta(t)}{2}\right]$$
$$= \rho d[2R \quad H\tan\theta(t)][y'(t)]\cos^2\theta(t)\frac{H\tan\theta(t)}{2}$$

$$M_{F_{\text{Part2+Part3}}} = 2\rho Hd[y'(t)]^2\sin^2\theta(t)\frac{H \quad 2h_c}{H}\left(\frac{H \quad 2h_c}{2} + h_c\right)$$
$$= 2\rho Hd[y'(t)]^2\sin^2\theta(t)\frac{H \quad 2h_c}{2}$$

and, thusly, the sum of the torques yield M_y:

$$M_y = 2\rho Hd[y'(t)]^2\sin^2\theta(t)\frac{H \quad 2h_c}{2}$$
$$+ \rho d[2R \quad H\tan\theta(t)][y'(t)]\cos^2\theta(t)\frac{H\tan\theta(t)}{2}$$

Similar analyses may be conducted to achieve all other component torques.

For the ease of analysis, the diatom frustule was simplified to a 2D model, as shown in Figure S1, but in in order to calculate different forces, we need to assign an effective "depth" to a diatom frustule, as shown in Figure S2b. In this figure, the two models need to be equivalent. To this end, we would like to make:

$$\begin{cases} \pi R^2 = 2Rd \\ \pi R^2 T + 2\pi RHT = 2RdT + 2dHT \end{cases}$$

so that the equivalent model will experience the same forces as a real diatom frustule. By solving the equations, we yield:

$$\begin{cases} d = \frac{\pi R}{2} \\ d = \frac{\pi R(R+2H)}{2(R+H)} \end{cases}$$

when H is much smaller than R, d can be considered as $\pi R/2$, which is suitable in our case.

DISPLACEMENT OF WATER SURFACE FROM RUPTURING OF DIFFERENT BUBBLES

In order to evaluate the agitations generated from the rupturing bubbles, COMSOL[1] simulations were conducted to simulate the rising and rupturing of an N_2 bubble. As shown in Figure S3, the vertical displacement of the water's surface is recorded 3 mm from

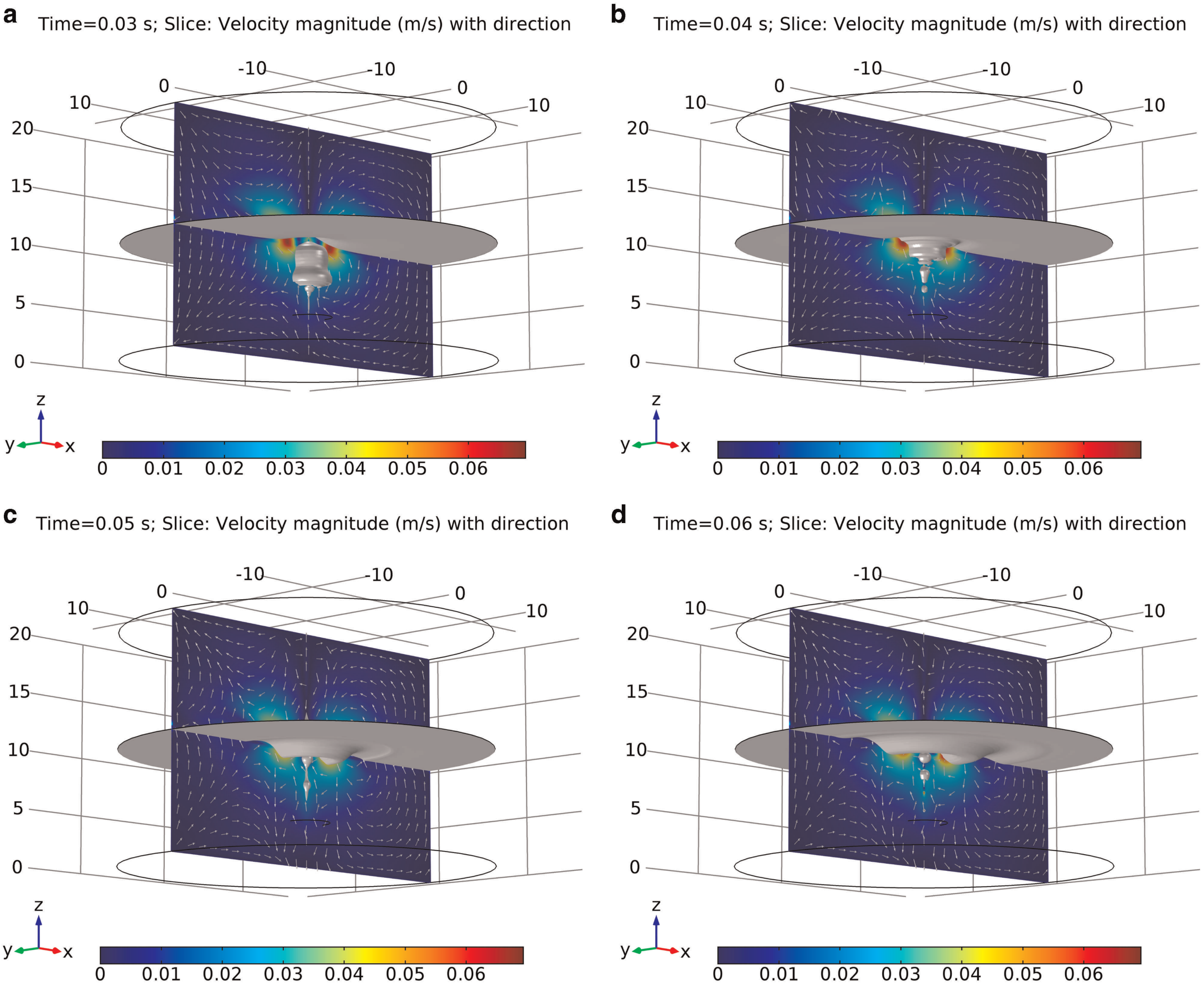

Figure S5 Velocity field of simulated rupturing bubble. Resultant "sinking flow" can be seen from the velocity's directions.

the center of the bubble. It is shown that the generated waves' magnitudes range from 0.7 mm to 2.5 mm, which is larger compared with the diameter of the diatoms.

In addition, it is shown in Figure S4 that the water jet is formed in the center of the rupturing bubble, which corresponds well with our experimental observations. Figure S5 comprehensively demonstrates the process of rupturing. Specifically, in Figure S5c and d, the flow field (grey arrows) indicates that there are sinking flows around the rupturing bubble. The simulation results jointly lend evidence to our hypothesis that under the influence of rupturing bubbles, diatom frustules were submerged under water.

REFERENCES

1 COMSOL AB. COMSOL Multiphysics® v. 5.2. COMSOL, 2015; www.comsol.com.
